# Alterations in global DNA methylation and hydroxymethylation are not detected in Alzheimer's disease

**DOI:** 10.1111/nan.12183

**Published:** 2015-04-23

**Authors:** Tammaryn Lashley, Priya Gami, Navid Valizadeh, Abi Li, Tamas Revesz, Robert Balazs

**Affiliations:** ^1^ Queen Square Brain Bank for Neurological DisordersDepartment of Molecular NeuroscienceUCL Institute of NeurologyLondonUK; ^2^ Reta Lila Weston Research Laboratories and Department of Molecular NeuroscienceUCL Institute of NeurologyLondonUK

**Keywords:** Alzheimer's disease, epigenetics, hydroxymethylation, methylation

## Abstract

**Aims:**

Genetic factors do not seem to account fully for Alzheimer disease (AD) pathogenesis. There is evidence for the contribution of environmental factors, whose effect may be mediated by epigenetic mechanisms. Epigenetics involves the regulation of gene expression independently of DNA sequence and these epigenetic changes are influenced by age and environmental factors, with DNA methylation being one of the best characterized epigenetic mechanisms. The human genome is predominantly methylated on CpG motifs, which results in gene silencing; however methylation within the body of the gene may mark active transcription. There is evidence suggesting an involvement of environmental factors in the pathogenesis of Alzheimer's disease (AD), which prompted our study examining DNA methylation in this disorder.

**Methods:**

Using immunohistochemistry with 5‐methylcytosine/5‐hydroxymethylcytosine antibodies we studied, in comparison with age matched controls, DNA methylation in sporadic and familial AD cases in the entorhinal cortex that exhibits substantial pathology and the cerebellum, which is relatively spared.

**Results:**

Neuronal nuclear labelling with 5‐methylcytosine (5mC) and 5‐hydroxymethylcytosine (5hmC) was evident in all cases studied. We did not detect any significant change in the levels of nuclear staining in the AD samples compared to neurologically normal controls. In the entorhinal cortex we also examined global DNA methylation and hydroxymethylation using an enzyme‐linked immunosorbent assay (ELISA).

**Conclusion:**

No significant differences were found between AD and control cases in global levels of 5mC and 5hmC in the entorhinal cortex using immunohistochemistry and enzyme‐linked immunosorbent assays.

## Introduction

Epigenetics is described as changes in gene expression, which do not involve alterations in the DNA sequence [1]. Such changes include DNA methylation, histone modifications and involve chromatin modifications. DNA methylation is one of the best characterized chromatin modifications and the human genome is predominantly methylated on CpG motifs. Most of the CpGs are clustered together (CpG islands). They are enriched in promoters, where their methylation results in gene silencing. However, the promoter methylation level is usually low and tissue‐specific methylation is primarily found at the shores of CpG islands [2]. Interestingly, methylation within the body of genes usually marks active transcription [3]. DNA methylation is mediated by DNA methyl transferases (DNMT), whereas the recently discovered 5‐hydoxymethyl deoxycytosine (5hmC) in DNA paved the way for the elucidation of the elusive 5mC demethylation pathway. The current view is that 5mC is oxidized by ten eleven translocation (TET) enzymes via 5hmC to 5‐formyl‐ and 5‐carboxylcytosine that are excised by a glycosidase and base‐excision repair introduces a cytosine to fill the gap (for review Song and He [4]). In addition to being an intermediate in demethylation, 5hmC and probably the further oxidation products also have a regulatory role, 5hmC marking gene activation. Epigenetic changes including DNA methylation/demethylation do take place during the life time, marking the influence of environmental factors on gene expression, thus they may mediate at the molecular level the effects of life events on disease risk and provide a potential explanation for disease discordance despite genetic similarities [5].

Compared with some other fields (e.g. cancer), epigenetic mechanisms have been studied less in Alzheimer's disease (AD), the most prevalent neurodegenerative disease, although there is now growing interest in such mechanisms [6]. The characteristic features of AD (cognitive impairment and neuropathological changes) are similar in the early‐onset and the late‐onset forms of the disease. Both forms of AD have genetic components. Although mutations in three genes (amyloid precursor protein (APP), presenilin‐1, and presenilin‐2), identified in the early‐onset familial form, account for only less than 2% of the cases, they have established the central role of amyloid in the pathogenesis of AD [7]. Large scale genome‐wide association studies (GWAS) have confirmed the role of ApoE as the most important genetic risk factor for late onset AD. In addition, they have also identified normal variants of further genes (*CLU*, *PICALM*, *BIN1*, *CR1*, *ABCA7*, *MS4A*, *EPHA1*, *CD33*, *CD2AP*), which are associated with increased disease risk [8]. Most recently *TREM2* variants with large effects have been recognized [9, 10]. However, because these risk factors are neither necessary nor sufficient for the development of late onset AD, other factors including epigenetic modifications are likely to play a role in the pathogenesis.

Interest in epigenetic changes in AD is currently increasing. Hitherto, most studies analysed DNA methylation in the brains of AD cases. Earlier studies examined DNA methylation in a few AD candidate genes and genes related to growth and development of the CNS and detected slight changes, if at all, compared with control brains [11, 12, 13, 14]. The study by Bakulski *et al*. was the first to provide a semi‐unbiased, quantitative, genome‐wide localization of DNA epigenetic differences in the human frontal cortex between AD cases and controls [15]. Compared with controls, 6% of genes featured on the arrayed were differentially methylated in the AD samples, while about the same number of sites being hypermethylated as hypomethylated. Therefore, in view of global changes, these studies on site‐specific DNA methylation showed rather modest alterations in AD compared with controls.

In contrast, Mastroeni *et al*. using immunocytochemistry reported a very severe reduction of this epigeneteic mark in AD entorhinal cortex [16] and the temporal cortex [17], while nuclear staining was similar to controls in the cerebellum that is relatively unaffected by AD pathology. These studies have been extended recently and a similar, although less severe, reduction in immunocytochemically detectable DNA methylation in the AD hippocampus was reported [18]. However, other studies have reported global DNA hypermethylation in various brain regions in AD cases compared to neurologically normal controls [19, 20, 21], whereas Condliffe *et al*. [22] found no significant difference in DNA methylation in the entorhinal cortex between AD and control cases. In some of these studies 5hmC levels were also determined by ELISA and immunohistochemically and these results are also controversial, Coppieters *et al*. reporting increased levels in AD brain tissue, whereas Chouliaras *et al*. and Condliffe *et al*. [18, 22] found decreases.

The discrepancies among these studies prompted us to screen a cohort of AD cases held at Queen Square Brain Bank for Neurological Disorders (UCL Institute of Neurology) and compare the levels of global DNA methylation and hydroxymethylation to neurologically normal control cases, to elucidate whether global DNA methylation is involved in the pathogenesis of AD.

## Material and methods

### Cases

The brain samples from AD and neurologically normal control cases were obtained from Queen Square Brain Bank (QSBB), UCL Institute of Neurology, London, UK. Ethical approval for the study was obtained from the National Hospital for Neurology and Neurosurgery Local Research Ethics Committee. AD patients fulfilled the clinical NINCDS criteria for probable AD [23] and met the neuropathological CERAD criteria for definitive AD [24]. Neurologically normal controls were cases that had died without history of dementia, psychiatric or neurological diseases. Detailed patient demographics are given in Table 1. The study was approved by the tissue request committee of the QSBB. A total of 12 AD cases and 14 neurologically normal controls (years, control: 75.93 ± 5.24 *vs.* AD: 68.17 ± 3.1; *P* = 0.2323) and post mortem delay (hours, control: 58.90 ± 10.75 *vs.* AD: 59.42 ± 8.1; *P* = 0.9701). All AD cases have been genetically tested and this study included two familial AD cases carrying a *PSEN1* intron 4 mutation (case 24) and an *APP V717L* mutation (case 25).

**Table 1:** Demographic data of AD and control cases

Case No.	Disease	Method	Age at death	Gender	Duration	PM delay (hours:minutes)	Braak and Braak	Aβ load (mature/diffuse)	pH
1	Control	IH, ELISA	38	M	n/a	80.35	0	0:0	5.87
2	Control	IH	82	F	n/a	72.00	3	1:2	6.03
3	Control	IH	71	M	n/a	38.50	0	0:0	6.30
4	Control	ELISA	69	M	n/a	171.00	1	1:2	6.63
5	Control	ELISA	99	F	n/a	32.05	4	2:2	6.23
6	Control	ELISA	92	F	n/a	87.50	3	1:3	6.50
7	Control	ELISA	87	F	n/a	51.40	1	0:1	6.17
8	Control	ELISA	95	F	n/a	39.00	4	2:2	6.02
9	Control	ELISA	82	F	n/a	91.20	2	1:3	6.25
10	Control	ELISA	93	F	n/a	29.40	3	2:3	6.43
11	Control	IH	73	F	n/a	24.00	2	1:2	5.72
12	Control	IH	68	F	n/a	45.05	0	0:0	6.91
13	Control	IH	34	M	n/a	14.00	0	0:0	n/a
14	Control	ELISA	80	F	n/a	49.10	2	0:0	5.79
15	Sporadic AD	ELISA	65	M	10	96.30	6	3:3	6.63
16	Sporadic AD	ELISA	86	F	16	90.20	6	3:3	6.22
17	Sporadic AD	ELISA	79	F	14	22.30	6	3:3	5.98
18	Sporadic AD	ELISA	79	M	16	64.55	5	2:3	6.40
19	Sporadic AD	ELISA	62	M	11	62.55	6	3:3	6.79
20	Sporadic AD	ELISA	81	M	12	78.15	6	3:3	6.49
21	Sporadic AD	IH, ELISA	66	F	14	51.20	6	3:3	6.20
22	Sporadic AD	IH, ELISA	62	F	11	16.00	6	3:3	6.31
23	Sporadic AD	IH	64	M	10	77.15	6	3:2	6.58
24	Sporadic AD	IH, ELISA	64	M	14	32.35	6	3:3	6.29
25	Familial AD (PSEN1 Intron 4)	IH	51	F	16	33.30	6	3:3	6.31
26	Familial AD (V717L APP)	IH	59	F	11	89.00	6	3:3	6.44

Neurological normal control and AD cases used in study. Cases were used for either immunohistochemistry (IHC) or enzyme‐linked immuno‐sorbent assay (ELISA). Neurofibrillary tangle pathology was staged according to the current pathological criteria (Braak and Braak) and Aβ load expressed according to the severity of the mature and diffuse plaques, where a score of 1 indicates mild deposition; 2 moderate and 3 severe Aβ deposition.

### Immunohistochemistry

Seven‐micron‐thick tissue sections from the entorhinal cortex and cerebellum were cut from the AD and control cases. Commercially available anti‐methylcytosine (1:500; Genway, San Diego, CA, USA) and anti‐hydroxmethylcytosine (1:10 000; Active Motiff, Belgium) antibodies were used in this study. Briefly, immunohistochemistry required pressure cooker pre‐treatment in citrate buffer pH6.0. Endogenous peroxidase activity was blocked with 0.3% H_2_O_2_ in methanol and non‐specific binding with 10% dried milk solution. Tissue sections were incubated with the primary antibodies overnight at 4°C, followed by biotinylated anti‐mouse IgG (1:200, 30 min; DAKO) and ABC complex (30 min; DAKO). Colour was developed with di‐aminobenzidine/H_2_O_2_ [25]. Deletion of primary antibody or incubation with pre‐immune serum resulted in abolition of specific immunoreactivity as previously reported [17]. Double immunohistochemical staining using anti‐methylcytosine and GFAP was carried out to confirm the majority of nuclei stained were neuronal. Briefly sections were incubated in both primary antibodies overnight at 4°C followed by incubation with Alexa Fluor 488 and 565 (1:1000, 2 h) and viewed with a Leica DM5500B fluorescence microscope using 3D deconvolution post‐processing.

### Semiquantification of immunohistochemical staining and statistical analysis

Semi‐quantitative immunohistochemical analysis was undertaken in both the AD and neurologically normal control cases. The region of interest was identified by comparing stained sections with haematoxylin and eosin (H&E) counterparts, on which a consultant neuropathologist identified the location of the layer 2/3 entorhinal cortex. Criteria were selected to identify neuronal nuclei and exclude glial nuclei. Using an objective ×40, only nuclei within a neurone with a diameter of at least 25 μm were counted; as this excluded oligodendrocytes, astrocytes and microglia, the procedure was considered to analyse neuronal nuclei. The observer was trained in identification of neuronal nuclei and distinguishing them from glial cell nuclei, and was blind to the underlying diagnosis in each case. Using a dictaphone, the staining intensity of the first 100 neuronal nuclei among the pre‐alpha clusters were recorded. Neuronal nuclei were divided into three levels on the basis of staining intensity: weak, medium and strong. Benchmark values for each of these intensities were established through collective review of stained specimens and arrival at consensus (Figure S1). To fairly assess differences in antibody staining between different cases inter and intra rateability analysis was performed. In order to establish that our method of discriminating nuclei based on staining intensity was reliable, and that our observations were reproducible and consistent, two observers carried out a preliminary count using six samples. An intra‐ and inter class correlation (ICC) validated this method by showing high concordance and low variability between observers (intra ICC 0.9713; inter ICC 0.9696). This method of analysis was undertaken to avoid bias due to neurone loss that occurs in AD.

### Quantification of global DNA methylation using ELISA

DNA was extracted from 100 mg of entorhinal cortex from 9 AD cases and 8 normal control cases (Table 1), using a phenol/chloroform extraction method [26]. Briefly frozen tissue samples were placed in 500 μl extraction buffer (0.5 % SDS, 0.1 M NaCl, 20 mM Trizma base, 25 mM EDTA disodium) along with 50 μl proteinase K (10 mg/mL). Samples were incubated at 55°C for 12 h, removed from the water bath and 0.8 mL PCI (phenol : chloroform : isoamyl alcohol; Sigma, Dorset, UK) added. The aqueous layer was removed from each sample and 50 μl of 3 M Na‐acetate was added together with 1 mL 100% ethanol. The samples were kept at −20°C for 2 h, spun at 12 000 rpm and the aqueous supernatant removed. The pellets were washed in ethanol and allowed to dry. Pellets were suspended in Tris‐EDTA buffer and concentration and quality determined using an Eppendorf spectrophotometer.

The 5mC and 5hmC content of the extracted DNA were measured by a methylated and hydroxymethylated DNA quantification kit (Epigentek, New York, USA). Briefly, wells were coated with 200 ng of DNA (each case was tested in duplicate). The methylated and hydroxymethylated fraction of DNA was detected using the respective capture and detection antibodies and quantified colorimetrically by reading the absorbance at 450 nm in a microplate reader. The results were expressed as units calculated according to the manufacturer's manual (5mC or 5hmC as a percentage of DNA). Multiple ELISAs were carried out on the same samples and an interclass correlation (ICC) validated this method by showing high concordance and low variability between different ELISA runs (inter ICC 0.8073).

### Statistical analysis

Statistical analysis was performed using Graphpad Prism 5 software (GraphPad Software Inc., CA, USA). Continuous variables were analysed using either a two‐tailed *t*‐test or a Mann–Whitney *U*‐test as appropriate, while continuous variables between several groups were compared using the Kruskal–Wallis test. The statistical significance level was established at *P* ≤ 0.05.

## Results

### Global DNA methylation and hydroxymethyation immunoreactivity

**Figure 1:**
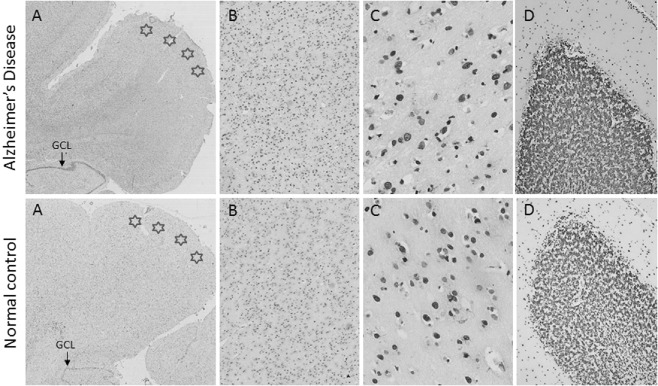
5‐methylcytosine immunohistochemistry in an AD (case 18) and a normal control (case 1). (**A**) 5‐methylcytosine immunohistochemistry in the entorhinal cortex (stars) demonstrates the amount of staining seen at three magnifications [×2 (**A**), ×10 (**B**) and ×40 (**C**)]. (**D**) Staining intensity in AD and control cerebellum (×10). Similar staining patterns were observed between the AD cases and normal controls.

For the immunohistochemical analysis of 5mC and 5hmC we examined 6 AD and 6 neurologically normal control cases from the cohort listed in Table 1 [age at death (years) − control: 61.00 ± 8.15 *vs.* AD: 61.00 ± 2.22 (*P* = 1.00)] and post mortem delay [(hours), control: 45.65 ± 10.68 *vs.* AD: 49.83 ± 11.56 (*P* = 0.7957)]. Using either the 5mC or 5hmC antibody, staining intensity of the neuronal nuclei showed variation that was evident in both the control and AD cases (Fig. 1 and Figures S1 and S2). 5mC and 5hmC immunohistochemistry staining of the cerebellum, a region relatively unaffected in AD, showed comparable staining patterns in both the neurologically normal controls and AD cases (Fig. 1 **D**). The variation in staining intensity was evident in all cell types and all neuronal and all glial nuclei were labelled, as no nuclei were observed which contained only the haematoxylin counterstain.

### Semi‐quantification of global DNA methylation and hydroxymethylation immunohistochemistry

**Figure 2:**
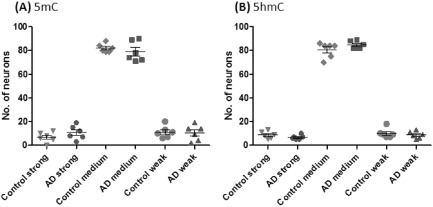
Semi‐quantitative analysis of global 5‐methylcytosine (**A**, 5mC) and 5‐hydroxymethylcytosine (**B**, 5hmC) immunohistochemistry in AD and normal controls. No significant difference were detected in numbers of strong, medium and weakly stained neuronal nuclei counted in the entorhinal cortex from AD *vs.* normal control cases.

In order to obtain semi‐quantitative data for analysis, we divided the different degrees of staining intensity of nuclei into three categories: weak, medium and strong (Figure S1). Neuronal nuclei were distinguished from glial nuclei by virtue of their larger size. This was also clarified using double immunohistochemical staining with GFAP (Figure S3). Based on the staining intensity, one hundred neuronal nuclei from the entorhinal cortex were assessed. No significant differences were detected between the AD and control cases in the degree of either the 5mC‐ or 5hmC‐labelled neuronal nuclei (5mC strong, *P* = 0.7525; medium, *P* = 0.5271; weak, *P* = 0.5625; 5hmC strong, *P* = 0.2575; medium, *P* = 0.4649; weak, *P* = 0.6863) (Fig. 2).

### Quantification of 5mC and 5hmC levels using enzyme‐linked immunosorbent assay (ELISA)

**Figure 3:**
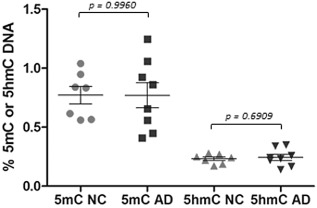
Levels of 5‐methycytosine (5mC) and 5‐hydroxymethylcytosine (5hmC) in AD and normal controls (NC). Amounts of 5mC and 5hmC were measured in the entorhinal cortex using ELISA in AD and normal controls. No statistical significance was found between the two groups.

**Figure 4:**
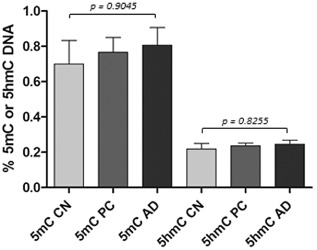
Levels of 5‐methycytosine (5mC) and 5‐hydroxymethylcytosine (5hmC) in AD, pre‐clinical (PC; clinically normal, but AD‐related pathology displaying group) and cognitively normal (CN, no AD‐related pathology) cases. Levels of 5mC and 5hmC were measured using ELISA. Kruskal–Wallis test showed no statistical significance among the three groups.

Cases used for the ELISA are listed in Table 1 [age at death (years), control: 81.67 ± 6.25 *vs.* AD: 71.56 ± 3.17 (*P* = 0.1683)] and post mortem delay [hours, control: 70.11 ± 14.86 *vs.* AD: 57.07 ± 9.65 (*P* = 0.4723)]. The quantification of the amount of 5mC and 5hmC showed no significant difference between the control and AD cases (*P* = 0.9960 and 0.6090 respectively) (Fig. 3). To assess whether underlying pathological lesions had an impact on DNA methylation, we reanalysed the data after dividing our cohort into three groups: (1) neurologically normal controls with no pathology (Braak and Braak stage 0), (2) neurologically normal controls with underlying Aβ and tau pathology (Braak and Braak stage 1–3) (‘preclinical AD’; CERAD scores and Braak and Braak staging listed in Table 1), (3) cases in which both the clinical symptoms and pathology fulfilled AD criteria (Braak and Braak stage 4–5). No significant difference in the amount of 5mC (*P* = 0.9045) and 5hmC (*P* = 0.8255) where seen when the cases were split according to the underlying pathology (Fig. 4).

### Correlation analysis between 5mC and 5hmC levels

**Figure 5:**
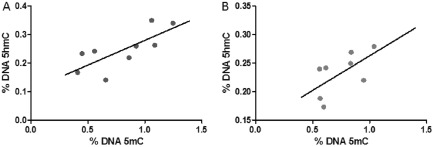
Pearson correlation analysis between 5‐methycytosine (5mC) and 5‐hydroxymethylcytosine (5hmC) levels in the entorhinal cortex in AD (**A**) and normal control cases (**B**). Levels of 5mC and 5hmC were significantly correlated in the AD cases. Although there was a trend in the normal controls this was not significant (*P* = 0.1017).

According to the current model, 5hmC is derived from 5mC by TET‐mediated oxidation [4]. Consistent with this, there was a positive, significant correlation between levels of 5mC and 5hmC in the AD cohort (Pearson correlation test: *r* = 0.7528; *P* = 0.0192), while the trend was also positive in the normal control cases, but this was not significant (*r* = 0.6192; *P* = 0.1017) (Fig. 5).

### Correlation between levels of 5mC/5hmC and other case variables

Pearson correlation tests were used to determine whether any of the case variables had an effect on the levels of 5mC or 5hmC. No significant correlation was found between the levels of 5mC or 5hmC and the age at death, Braak and Braak tau stages, and total Aβ plaque load (Table S1).

## Discussion

Global DNA methylation was determined in the present work in the entorhinal cortex of AD cases and controls using immunohistochemistry with 5mC antibody. The staining was primarily in nuclei and every nucleus was labelled. We counted only nuclei the diameter of which was at least 25 μm, and these were considered as neuronal. This analysis eliminated the bias that could be due to neurone cell loss in AD. Differences were observed in the staining intensity; therefore, we adopted a semi‐quantitative approach in order to generate data we could test for statistical significance, dividing stained neuronal nuclei into three categories of intensity (weak, medium, strong). No significant difference was observed between the degree of labelling between AD and control cases in the cohort of cases used in this study.

We also determined global DNA methylation using an enzyme‐linked immunosorbent assay and observed no significant differences between AD and control cases. The neuropathological examination showed that some of the clinically normal cases had AD associated pathology, amyloid plaques and neurofibrillary tangles in the brain (defined here as ‘preclinical AD’). These cases were separated from the control group and the data were reanalysed comparing the clinically and pathologically normal control group with both the ‘preclinical’ and the patent AD cohorts. No significant difference was detected in comparison with control, in contrast to the report by Bradley‐Whitman and Lovell *et al*., which showed increased DNA methylation not only in late onset AD, but also in the preclinical subset of cases [20].

Demethylation of 5mC in DNA is mediated by TET‐catalysed oxidation to 5hmC that, in addition of being an intermediate in the demethylation pathway, has also regulatory functions [4]. Immunohistochemical detection of 5hmC showed, similar observations to the 5mC labelling, that all nuclei were stained and no significant differences were detected between the labelling intensity of AD and control cases. This observation was confirmed by determining 5hmC content using ELISA, which also showed no significant differences between AD and control cases. There was significant correlation between 5mC and 5hmC levels in the AD group and a positive, although no significant trend was observed in the control group. These observations are consistent with the findings of Chouliaras *et al*. and Coppieters *et al*. [18, 21].

Using Pearson correlation analysis, we observed no significant effects between DNA modifications and case variables (age, Braak and Braak tau staging and total Aβ levels). This is in agreement with the observations of Coppieters *et al*., who also found that the relation between DNA modifications and post mortem delay is not significant [21]. It has, however, been reported that 5mC and 5hmC levels increase with age [18, 27]. As the major increase in 5mC levels occurs between early development and about 60 years [27] and our samples with the exception of a few were relatively old (see Table 1), this might explain the absence of correlation with age [21].

Our observation that global DNA methylation is not significantly different in the entorhinal cortex of AD cases from controls is consistent with the finding of Condliffe *et al*. [22]. Further, genome site‐specific analysis of DNA methylation has failed to identify marked alterations in the AD brain. Earlier studies, examining DNA methylation of a few AD candidate genes detected slight changes, if at all, compared to control brains [11, 12, 13]. In a quantitative, genome‐wide localization of DNA methylation differences in the human frontal cortex between AD cases and control, Bakulski *et al*. found bidirectional changes with about the same number of hypermethylation as hypomethylation sites [15].

In contrast, Mastroeni *et al*. used 5mC immunocytochemistry and reported a very severe reduction of this epigenetic mark in the AD entorhinal cortex [16, 17]. These studies have been extended recently and a reduction in immunocytochemically detected DNA methylation in the AD hippocampus was reported [18]. A decrease in 5hmC levels in the AD hippocampus was also reported in this work, which was also found by Condliffe *et al*., who also detected reduced 5hmC levels in the cerebellum that is relatively unaffected in AD [22]. However, other observations show an increase in global DNA methylation in the AD brain and some of these studies also found an increase in the level of 5hmC in the AD brain [19, 20, 21] (Table S2).

The reason for the differences in the reported DNA methylation in AD is not clear. Different brain regions were studied in the various works and there is evidence that methylated/hydroxymethylated DNA levels vary among brain regions [28, 29, 30]. However, some of the findings by the different groups are contradictory even for the same region. There have also been differences in the tissue processing and immunostaining procedures. Further, the sample size in many of these studies, including our own, was relatively small.

The limitation of the global DNA methylation approach should be kept in mind. Site‐specific differential DNA methylation has been already shown in AD and might have an important bearing on disease pathology. Due to the heterogeneous cellular composition of the brain, cell‐type specific investigations are of paramount importance. Further studies are needed in which use is made of current technologies, including genome‐wide analysis of specific cell types, to appreciate the involvement of DNA methylation in AD pathology.

## Author contributions

RB initiated the study. TL, NV and TR selected the cases and undertook the pathological analysis; PG and AL ran the ELISA experiments. TL and RB wrote the paper and all authors were involved in editing the manuscript.

## Supporting information


**Figure S1.** 5‐methylcytosine immunohistochemical staining demonstrating the three levels of neuronal nuclei staining intensity; weak, medium and strong. Benchmark values for each of these intensities were established through collective review of stained specimens and arrival at consensus.
**Figure S2.** 5‐hydroxymethylcytosine immunohistochemistry in an AD (case 18) and normal control (case 1). Demonstrating 5hmC staining in the entorhinal cortex of AD cases and normal controls.
**Figure S3.** 5‐Methylcytosine (green) and GFAP (red) double immunohistochemical staining in the entorhinal cortex. The majority of nuclei stained with the 5‐methylcytosine antibody are not labelled with GFAP antibody. Arrows show astrocytes that are both methylcytosine and GFAP positive. Higher magnification is shown in the inserts.
**Table S1.** Pearson correlation analysis to determine whether case variables correlated with the levels of 5mC and 5hmC as measured by ELISA. No significance was found between age at death, Braak and Braak tau staging and Aβ load in comparison with either 5mC and 5hmC levels.
**Table S2.** Summary of recent findings on DNA methylation in the brain in AD. *In AD cases accelerated ageing‐related changes in 2 genes from 50 determined; ** Age‐dependent epigenetic drift is more pronounced in AD than in control cases. IHC, immunohistochemistry. 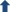 hypermethylation in AD; 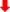 hypomethylation in AD; ‘0’ no significant difference between controls and AD cases.
