# Supplementary material for: Alterations in global DNA methylation and hydroxymethylation are not detected in Alzheimer's disease
Source: Neuropathol Appl Neurobiol. 2015 Apr 23;41(4):497–506. doi: 10.1111/nan.12183 (PMC4879505; doi:10.1111/nan.12183)
Supplement: Supplementary file 1 — Figure S1. 5‐methylcytosine immunohistochemical staining demonstrating the three levels of neuronal nuclei staining intensity; weak, medium and strong. Benchmark values for each of these intensities were established through collective review of stained specimens and arrival at consensus. Figure S2. 5‐hydroxymethylcytosine immunohistochemistry in an AD (case 18) and normal control (case 1). Demonstrating 5hmC staining in the entorhinal cortex of AD cases and normal controls. Figure S3. 5‐Methylcytosine (green) and GFAP (red) double immunohistochemical staining in the entorhinal cortex. The majority of nuclei stained with the 5‐methylcytosine antibody are not labelled with GFAP antibody. Arrows show astrocytes that are both methylcytosine and GFAP positive. Higher magnification is shown in the inserts. Table S1. Pearson correlation analysis to determine whether case variables correlated with the levels of 5mC and 5hmC as measured by ELISA. No significance was found between age at death, Braak and Braak tau staging and Aβ load in comparison with either 5mC and 5hmC levels. Table S2. Summary of recent findings on DNA methylation in the brain in AD. *In AD cases accelerated ageing‐related changes in 2 genes from 50 determined; ** Age‐dependent epigenetic drift is more pronounced in AD than in control cases. IHC, immunohistochemistry. hypermethylation in AD; hypomethylation in AD; ‘0’ no significant difference between controls and AD cases. [file NAN-41-497-s001.zip › NAN_12183_supp0004-Supplementary table 1.docx]

**Supplementary table 1:**

| **Variable** | **% DNA 5mC** | | **% DNA 5hmC** | |
| --- | --- | --- | --- | --- |
|  | *r value* | *p value* | *r value* | *p value* |
| Age at death | *-0.1995* | *0.4428* | *-0.1745* | *0.5030* |
| Braak and Braak Tau staging | *0.1218* | *0.614* | *0.1002* | *0.7020* |
| Aβ load | *0.2089* | *0.4210* | *0.1597* | *0.5405* |
